# Supplementary material for: Tissue-Specific Transcriptome Analysis Reveals Candidate Transcripts Associated with the Process of Programmed B Chromosome Elimination in Aegilops speltoides
Source: Int J Mol Sci. 2020 Oct 14;21(20):7596. doi: 10.3390/ijms21207596 (PMC7593951; doi:10.3390/ijms21207596)
Supplement: Supplementary file 1 [file ijms-21-07596-s001.zip › SUPPL FINAL/ijms-949070-Supplementary Materials.pdf]

International Journal of
Molecular Sciences

MDPI

Supplementary Materials

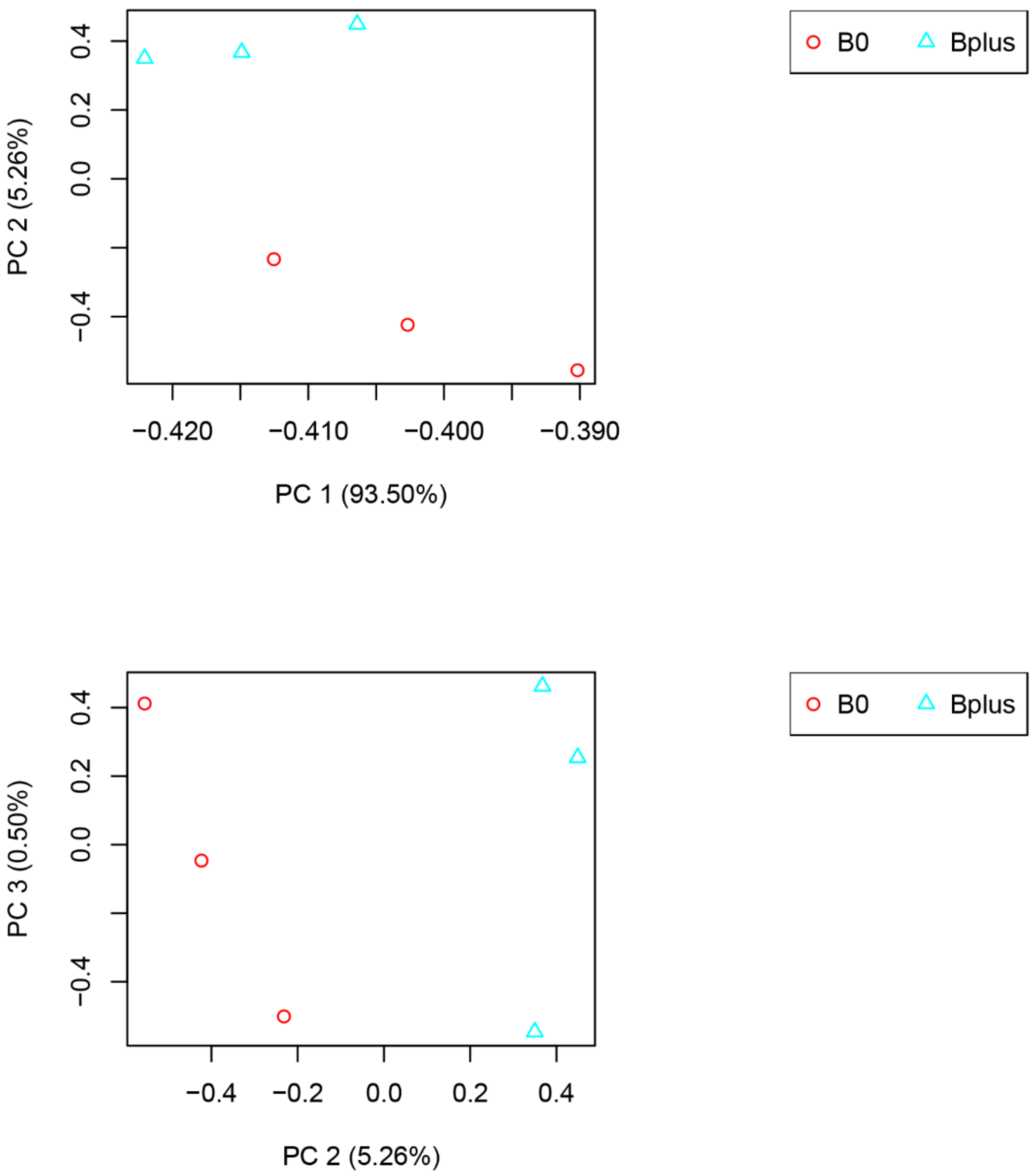

Figure S1. Assessing variability of biological replicates and relationship among samples without B chromosomes (B0) and with eliminating B chromosomes (Bplus). Principal Component Analysis (PCA) plots displaying variability within the 6 samples along PC1 and PC2 as well as PC2 and PC3.

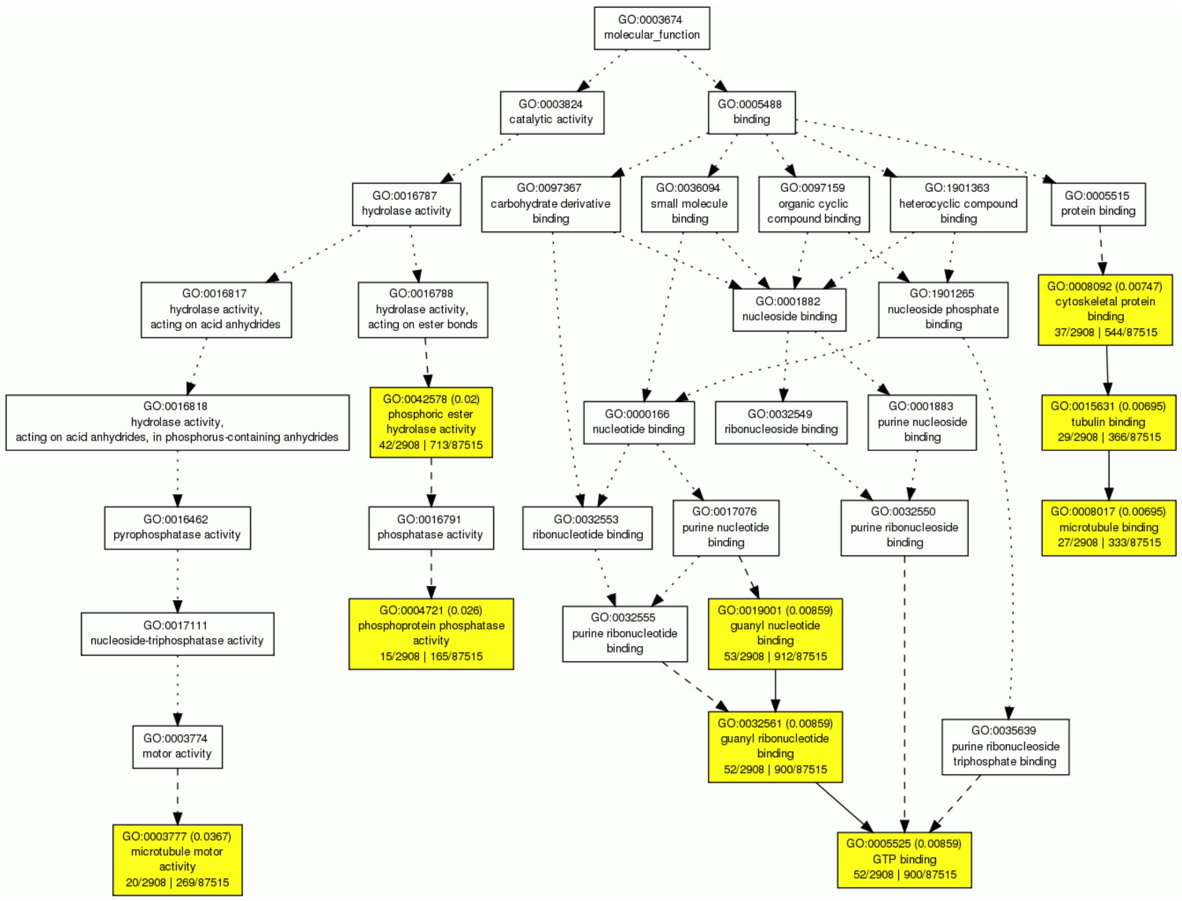

Figure S2. GO term enrichment analysis for genes with statistically significant up-regulated changes during B chromosome elimination. A SEA tool of the AgriGo v2.0 displays hierarchical tree graphs of statistically significant GO terms (adjusted *p*-value < 0.05). in molecular function category. The significant terms are colored, while non-significant terms are shown as white boxes. The type of lines (solid, dashed, and dotted) reflects two, one and zero enriched terms at both ends connected by the line, respectively. The information within each box of the GO term includes GO term, adjusted *p*-value, description of the term, item number corresponding to the GO term in the query list and background, and the total number of query list and background.

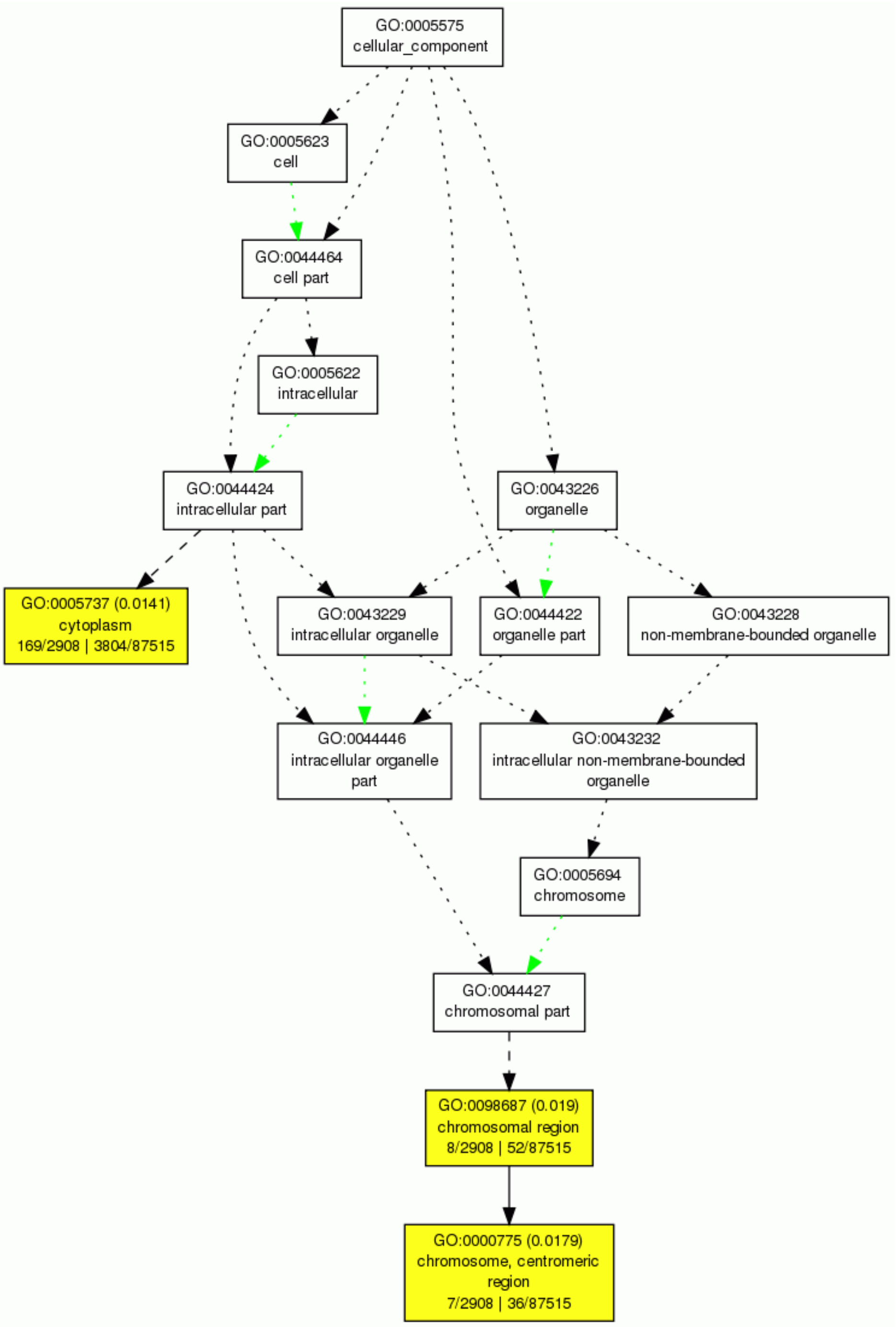

Figure S3. GO term enrichment analysis for genes with statistically significant up-regulated changes during B chromosome elimination. A SEA tool of the AgriGo v2.0 displays hierarchical tree graphs of statistically significant GO terms (adjusted *p*-value < 0.05) in cellular compartment category. The significant terms are colored, while non-significant terms are shown as white boxes. The type of lines (solid, dashed, and dotted) reflects two, one and zero enriched terms at both ends connected by the line, respectively. Arrow in green describes negative regulation. The information within each box of the GO term includes GO term, adjusted *p*-value, description of the term, item number corresponding to the GO term in the query list and background, and total number of query list and background.

Table S1. Primers used for PCR and RT-qPCR.

Table S2. Summary statistics and quality assessment for the *de novo* assembly based on Trinity and Transrate.

Table S3. Results of Gene Set Enrichment Analysis (GSEA, AgriGo v2. analytical toolkit) using transcript isoforms up-regulated during the B chromosome elimination in *Ae. speltoides*. Background reference for the enrichment analysis is the wheat genome.

Table S4. Transcript isoforms up-regulated during the B chromosome elimination in *Ae. speltoides* and representing the enriched term "microtubule-based process" based on AgriGo v2. Background reference for the enrichment analysis is wheat genome.

Table S5. Transcript isoforms up-regulated during the B chromosome elimination in *Ae. speltoides* and representing the enriched term "chromosomal region" based on AgriGo v2. Background reference for the enrichment analysis is wheat genome.

Table S6. Results of Gene Set Enrichment Analysis (GSEA, AgriGo v2. analytical toolkit) using transcript isoforms down-regulated during the B chromosome elimination in *Ae. speltoides*. Background reference for the enrichment analysis is wheat genome.

Table S7. Transcript isoforms downregulated during the B chromosome elimination and representing the enriched term "cellular response to stress" based on AgriGo v2. Background reference for the enrichment analysis is wheat genome.

Table S8. Results of differentially expression analysis in embryos of *Ae. speltoides* based on DESeq2. Annotated transcript isoforms uniquely expressed during the B chromosome elimination are shown.

Table S9. Tissue sections isolated by laser capture microdissection.
