# Supplementary figures and images for: Tissue-Specific Transcriptome Analysis Reveals Candidate Transcripts Associated with the Process of Programmed B Chromosome Elimination in Aegilops speltoides
Source: Int J Mol Sci. 2020 Oct 14;21(20):7596. doi: 10.3390/ijms21207596 (PMC7593951; doi:10.3390/ijms21207596)

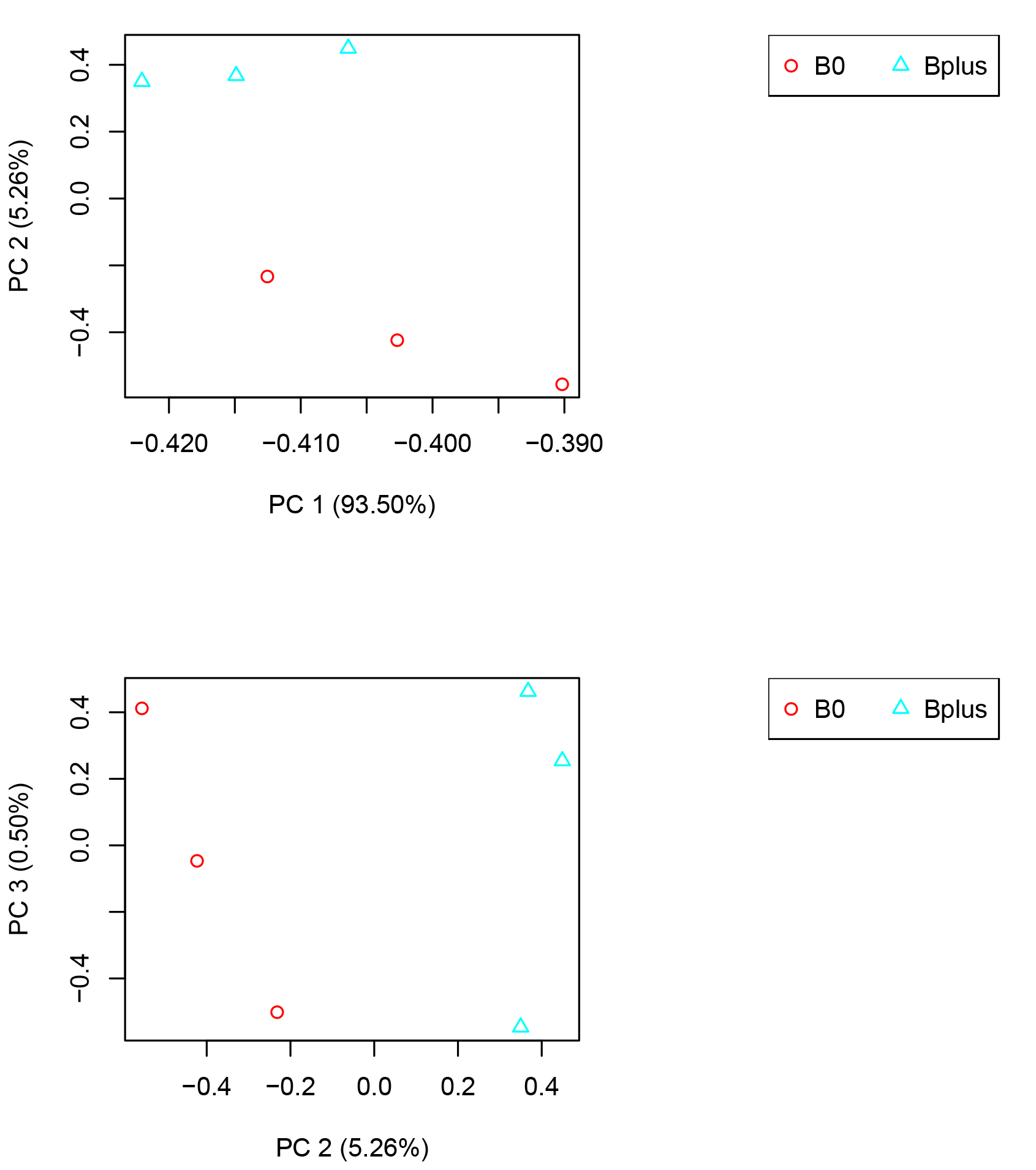

Supplement: Supplementary file 1 [file ijms-21-07596-s001.zip › SUPPL FINAL/Figure S1.png]

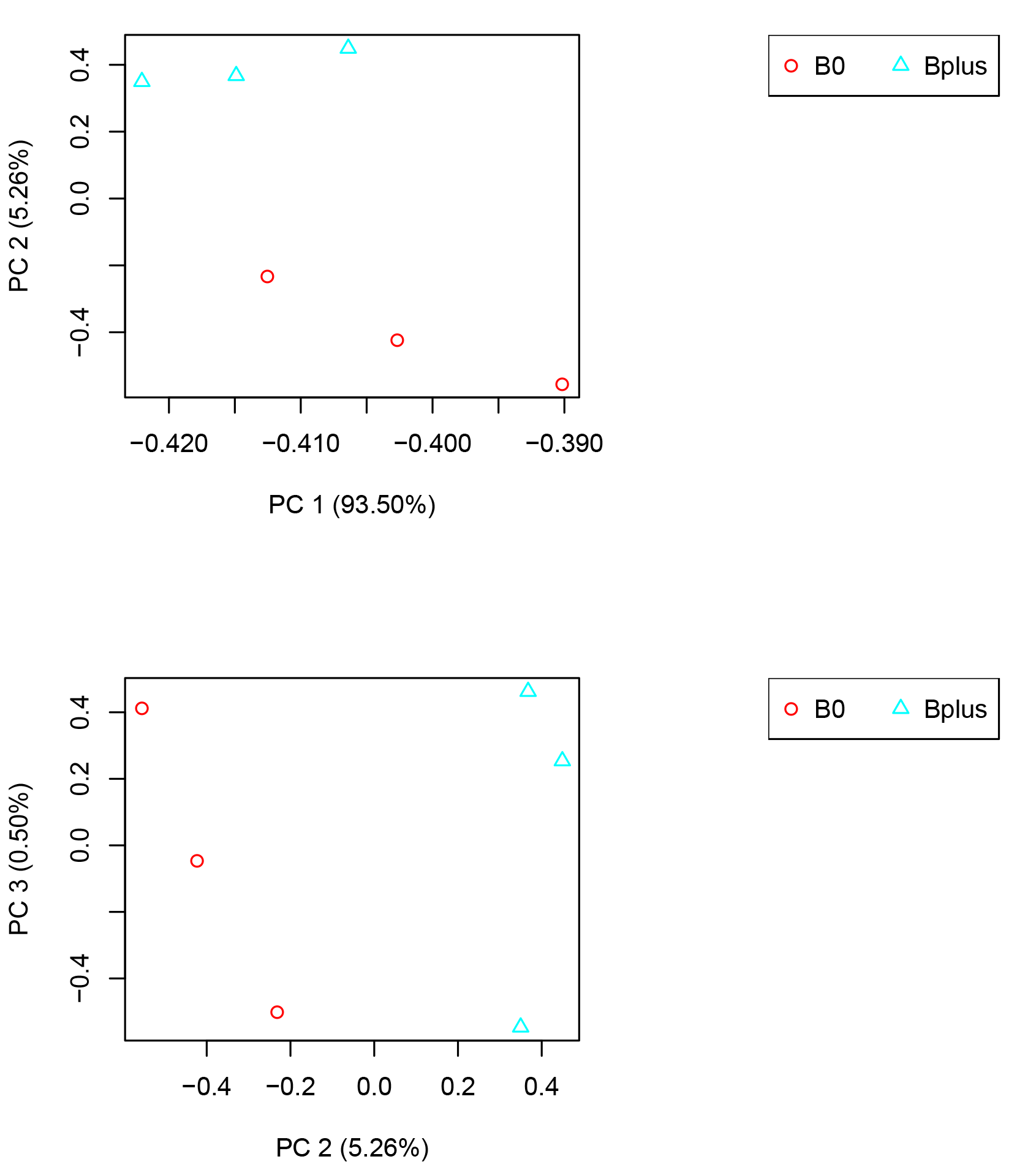

Supplement: Supplementary file 1 [file ijms-21-07596-s001.zip › SUPPL FINAL/Figure S1.tif]

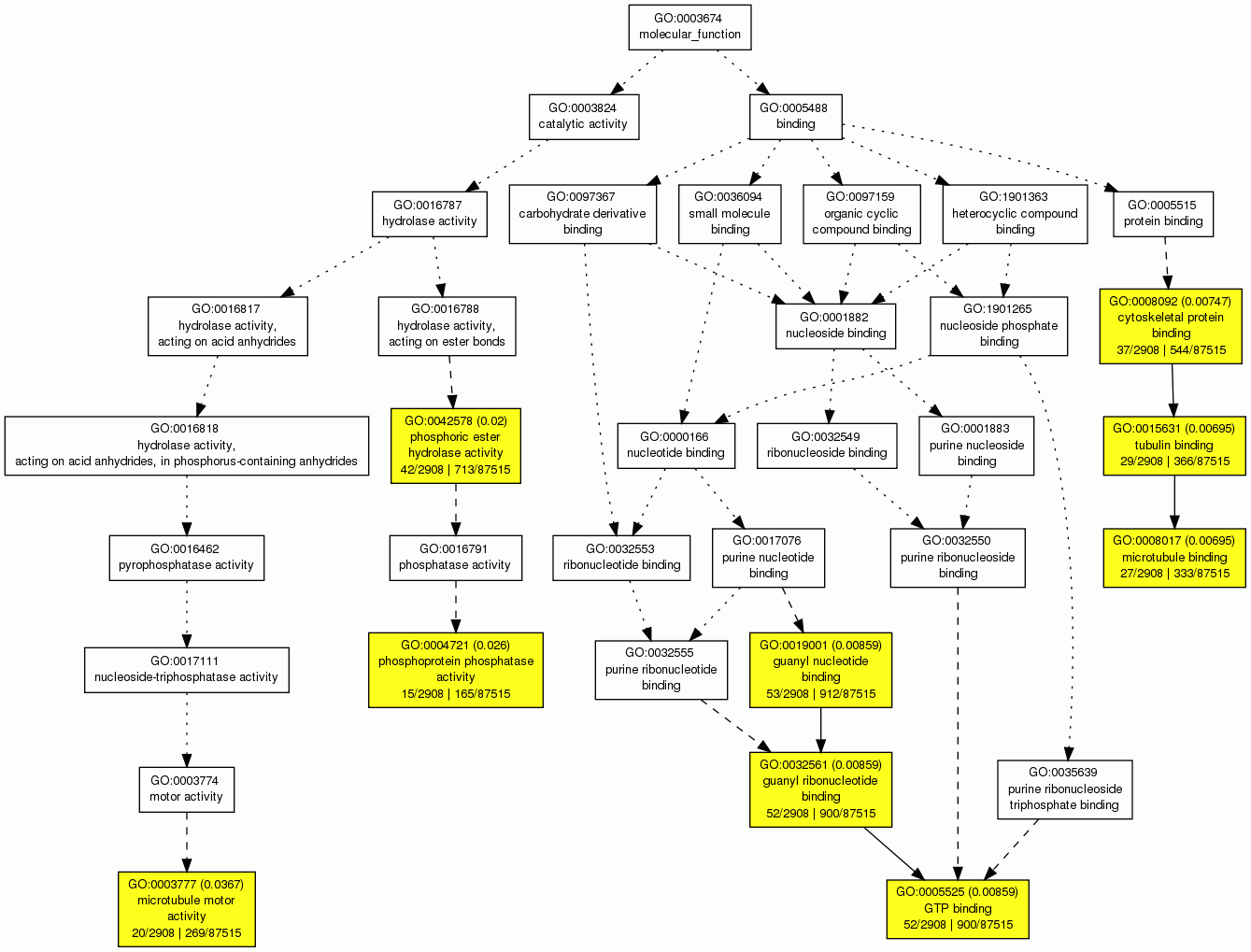

Supplement: Supplementary file 1 [file ijms-21-07596-s001.zip › SUPPL FINAL/Figure S2.gif]

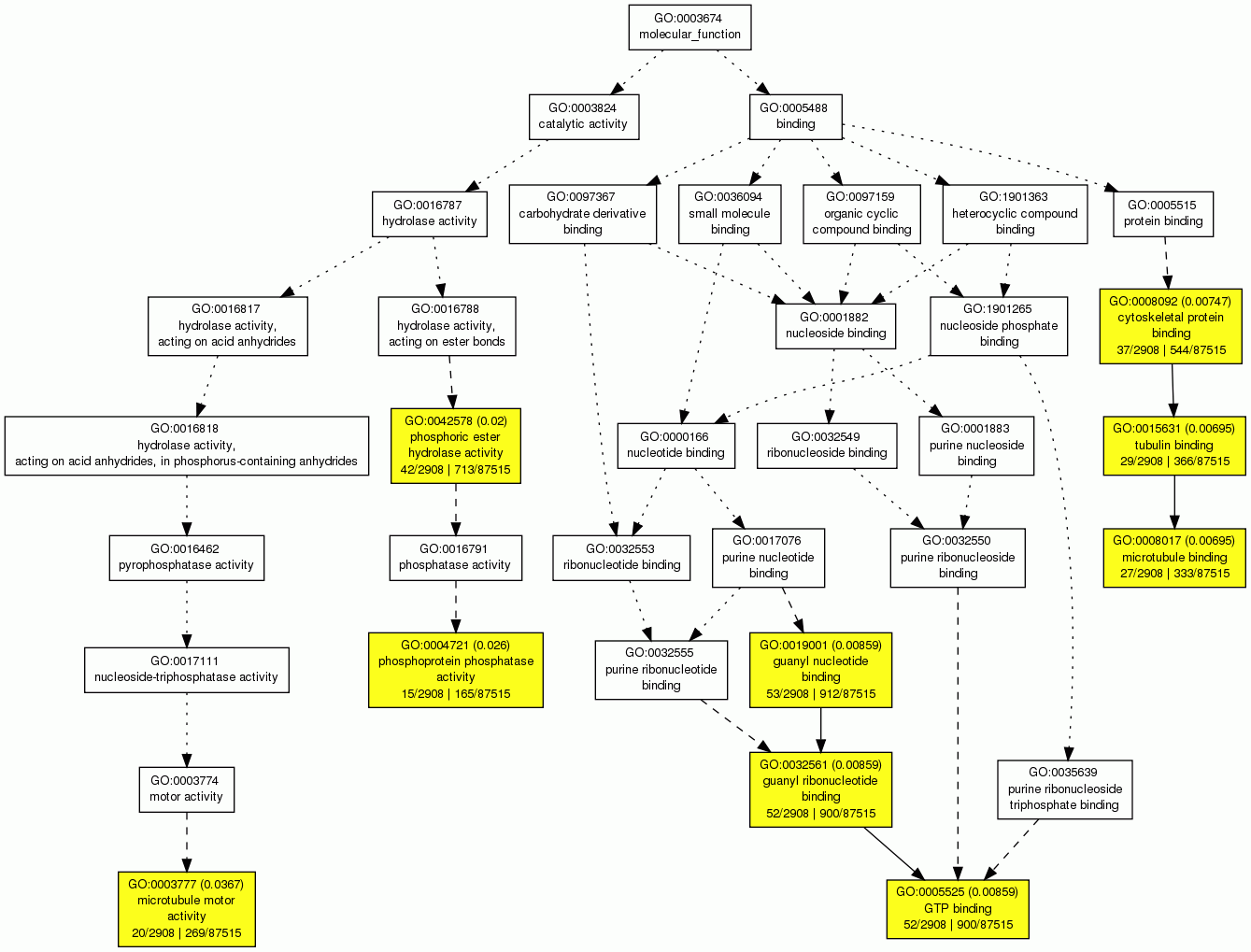

Supplement: Supplementary file 1 [file ijms-21-07596-s001.zip › SUPPL FINAL/Figure S2.png]
